# Correlations between intraretinal cystoid cavities and pre- and postoperative characteristics of eyes after closure of idiopathic macular hole

**DOI:** 10.1038/s41598-020-59295-7

**Published:** 2020-02-11

**Authors:** Kensuke Goto, Takeshi Iwase, Kentaro Yamamoto, Eimei Ra, Hiroko Terasaki

**Affiliations:** Department of Ophthalmology, Nagoya University Graduate School of Medicine, Nagoya, Japan

**Keywords:** Medical imaging, Outcomes research

## Abstract

Intraretinal cystoid cavities have been detected at the edges of macular holes (MHs) but their clinical characteristics and their relationship to the MH variables have not been determined. We measured the areas of the intraretinal cystoid cavity in 111 eyes with MHs in the OCT images preoperatively. Our results showed that the intraretinal cystoid cavities were located in the Henle fiber layer-outer nuclear layer (HFL-ONL) complex in 106 eyes and in the inner nuclear layer (INL) in 89 eyes. All were resolved after the initial vitrectomy to close the MH. The mean area of the cystoid cavity was greater in the HFL-ONL complex (55.9 ± 42.7 × 10^3^ μm^2^) than in the INL (9.1 ± 9.8 × 10^3^ μm^2^; *P* < 0.001). The area of the cystoid cavities was significantly correlated with the basal MH size (*r* = 0.465,*P* < 0.001), the external limiting membrane height (*r* = 0.793, *P* < 0.001), and the maximum retinal thickness (*r* = 0.757, *P* < 0.001). The area of the cystoid cavities was significantly correlated with the preoperative best-corrected visual acuity (BCVA; *r* = 0.361, *P* < 0.001), but not with the postoperative BCVA or the integrity of any of the outer retinal microstructural bands. The presence of intraretinal cystoid cavities was related to some morphological characteristics, but not to the postoperative BCVA or the restoration of the outer retinal bands.

## Introduction

An idiopathic macular hole (MH) is found more commonly in the eyes of middle-aged and older individuals and can cause metamorphopsia and a decrease of the best-corrected visual acuity (BCVA). There are several suggestions on the mechanisms causing the formation of a full-thickness MH. Gass suggested that a MH begins by a tangential traction of the perifoveal vitreous cortex on the foveal retina which results in a dehiscence that progresses to a full-thickness MH^1,2^.

Optical coherence tomography (OCT) has enabled clinicians to evaluate the foveal microstructures in eyes with a MH. Recent studies have determined that a MH is initially formed by a perifoveal posterior vitreous detachment (PVD) which causes an anteroposterior vitreomacular traction (VMT)^3–5^.

Intraretinal cystoid cavities at the edges of a MH have been recognized in the images obtained by the early OCT devices^6^. These intraretinal hyporeflective spaces, the intraretinal cystoid cavities, are frequently observed around the edges of MHs^7^. However, the area of intraretinal cystoid cavities varies among eyes with a MH, and the clinical features of these eyes have not been quantified.

Recent advances in the surgical techniques for closing a MH have improved the anatomical closure rates and the postoperative BCVAs. However, even after a successful closure of a MH, the postoperative BCVA can be unsatisfactory in some cases. To predict the postoperative BCVA, several preoperative OCT features such as the basal and minimum MH size^8,9^ and the maximum retinal thickness^10^ have been evaluated. Several investigators have reported that the degree of disruption of the microstructures of the photoreceptors in the preoperative and postoperative OCT images were significantly correlated with the postoperative BCVA in eyes with a MH^11,12^. Although the presence of intraretinal cystoid cavities is strongly related to the morphological changes at the edges of a MH, there have been only a few studies on the relationship between the characteristics of the cystoid cavities and the pre- and postoperative BCVA^13^.

Thus, the purpose of this study was to determine the clinical characteristics of intraretinal cystoid cavities and their correlations with the MH variables, and also to determine whether the presence of intraretinal cystoid cavities is correlated with the pre- or postoperative BCVA and the morphology of the outer retina.

## Results

### Demographics of patients

One hundred sixty-five eyes of 165 patients with a MH underwent vitrectomy with the internal limiting membrane (ILM) peeling between January 2013 and June 2018, and the MH was successfully closed after the initial surgery in all eyes. Of the 165 eyes, 54 were excluded; 36 for high myopia, 12 for other retinal diseases, 5 for past intraocular surgery, and 1 for prior trauma. In the end, 111 eyes of 111 patients (mean age, 66.2 ± 6.2 years) were studied.

The demographics of the patients and the results of the measurements are shown in Table 1. The number of eyes with MH stage 2 was 42, stage 3 was 30, and stage 4 was 39. The number of eyes that did not have an intraretinal cystoid cavity was 3 for the Stage 2 group and 2 for the Stage 4 group. The preoperative BCVA was 0.62 ± 0.25 logarithm of the minimum angle of resolution (logMAR) units, and it was significantly improved to 0.20 ± 0.17 logMAR units after the surgery (*P* < 0.001).

**Table 1:** Clinical characteristics of subjects.

Characteristics	n = 111
Age (year)	66.3 ± 6.2
Male: female (patients)	41:70
Right: Left (eyes)	55:56
Axial length (mm)	23.9 ± 1.3
Duration of symptom (month)	3.4 ± 2.6
MH stage 2: 3: 4 (eyes)	42:30:39
**Area of intraretinal cystoid cavities**	
in INL (×10^3^ μm^2^)	9.1 ± 9.8
in HFL-ONL complex (×10^3^ μm^2^)	55.9 ± 42.7
in all (×10^3^ μm^2^)	65.0 ± 46.6
Basal MH size (μm)	655 ± 250
Minimum MH size (μm)	281 ± 165
Basal MH size -minimum MH size (μm)	374 ± 172
Maximum retinal thickness (μm)	404 ± 90
ELM height (μm)	177 ± 50
Angle of fluid cuff (°)	35.5 ± 7.5
ELM defect length (μm)	406 ± 220
EZ defect length (μm)	431 ± 251
CIZ defect length (μm)	938 ± 733
Preoperative BCVA (logMAR)	0.62 ± 0.25
Postoperative BCVA (logMAR)	0.20 ± 0.17
PPV + PEA + IOL/PPV (eyes)	95:16
Tamponade, Air: C_3_F_8_:SF_6_ (eyes)	7:39:65

MH: macular hole, INL: inter nuclear layer, HFL-ONL: Henle fiber layer-outer nuclear layer, ELM: external limiting membrane, EZ: ellipsoid zone, CIZ: cone interdigitation zone, BCVA: best-corrected visual acuity, logMAR: logarithm of minimum angle of resolution, PPV: pars plana vitrectomy, PEA: phacoemulsification and aspiration, IOL: intra-ocular lens, C3F8: Octafluoropropane, SF6: Sulfur hexafluoride

### Characteristics of intraretinal cystoids cavities

At least one intraretinal cystoid cavity was observed in 106 of 111 eyes (95.5%) preoperatively, and none was observed in all eyes postoperatively at 12 weeks. Of the 106 eyes, intraretinal cystoid cavities were observed in the Henle fiber layer-outer nuclear layer (HFL-ONL) complex in all of the eyes and in the inner nuclear layer (INL) in 89 eyes. The area of the intraretinal cystoid cavity in the preoperative OCT images was 9.1 ± 9.8 × 10^3^ μm^2^ in the INL and 55.9 ± 42.7 × 10^3^ μm^2^ in the HFL-ONL complex. The cavities were significantly larger in the HFL-ONL complex than in the INL (*P* < 0.001).

### Correlations between area of intraretinal cystoid cavity and morphology of functional variables

**Figure 1:**
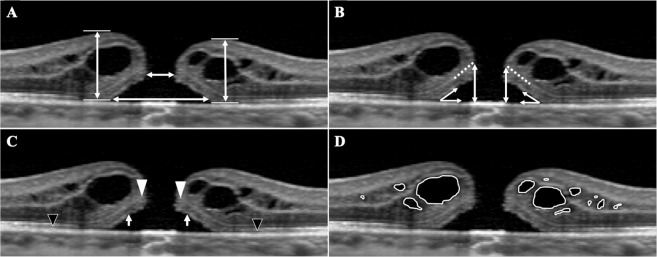
Measurements of the morphological characteristics of a macular hole (MH) in the optical coherence tomographic (OCT) images of eyes with an idiopathic MH. The minimum MH size (shorter horizontal line), the basal MH size (longer horizontal line), and the maximum retinal thickness (vertical lines) were measured by placing a tangential line across the highest points on both side of the hole, and then drawing a perpendicular line to the RPE (**A**). A line passing through the external limiting membrane (ELM) within the fluid cuff was extended to the edge of the MH (dotted-line), and the distance between the extended ELM edge and the RPE was defined as the ELM height (long arrows). The angle of fluid cuff was also measured at the both sides of MH (**B**). The distance between each edges of the ELM (the distance between white arrowheads), the EZ (the distance between long arrows), and the CIZ (the distance between black arrowheads), were defined as the length of the defect (**C**). The area of a intraretinal cystoid cavity was measured with ImageJ software by manually tracing the outline of the cystoid cavities (**D**).

**Figure 2:**
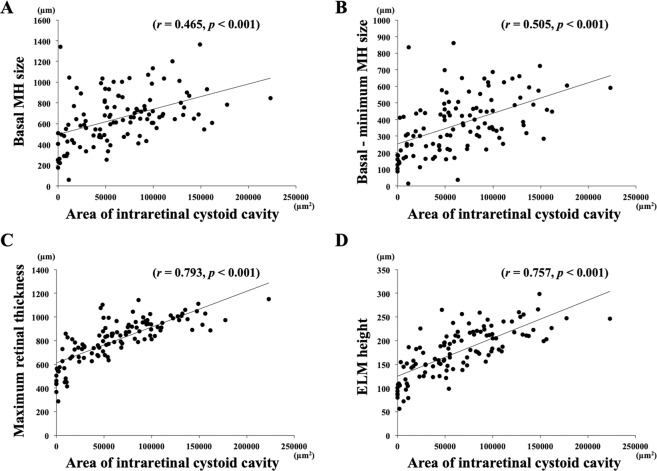
Correlations between area of the intraretinal cystoid cavity and the other variables. The area of intraretinal cystoid cavity was significantly and positively correlated with the basal MH size (*r* = 0.465, *P* < 0.001), the distance from basal to minimum MH size (*r* = 0.505, *P* < 0.001), the maximum retinal thickness (*r* = 0.793, *P* < 0.001), and the ELM height (*r* = 0.757, *P* < 0.001).

The area of cystoid cavity was significantly correlated with the basal MH size (*r* = 0.465, *P* < 0.001), with the difference between the basal and minimum MH size (*r* = 0.505, *P* < 0.001), with the vertical height of the central external limiting membrane (ELM) above the retinal pigment epithelium (ELM height) (*r* = 0.793, *P* < 0.001), with the maximum retinal thickness (*r* = 0.757, *P* < 0.001), with the length of the ELM defect (*r* = 0.298, *P = *0.003), with the length of the ellipsoid zone (EZ) defect (*r* = 0.312, *P = *0.002), and with the length of the cone interdigitation zone (CIZ) defect (*r* = 0.499, *P* < 0.001; Table 2; Figs. 1, 2).

**Table 2:** Results of Pearson’s correlation coefficient between area of intraretinal cystoid cavity and other preoperative variables.

Variables	*r*	*p*-value
Age (year)	−0.036	0.709
Axial length (mm)	−0.030	0.753
Preoperative BCVA (log MAR)	0.404	<0.001
Duration of symptom (months)	−0.126	0.186
Basal MH size (μm)	0.465	<0.001
Minimum MH size (μm)	0.167	0.080
Basal - minimum MH size (μm)	0.505	<0.001
Maximum retinal thickness (μm)	0.793	<0.001
ELM height (μm)	0.757	<0.001
Angle of fluid cuff (°)	0.049	0.610
ELM defect length (μm)	0.298	0.003
EZ defect length (μm)	0.312	0.002
CIZ defect length (μm)	0.499	<0.001

**Figure 3:**
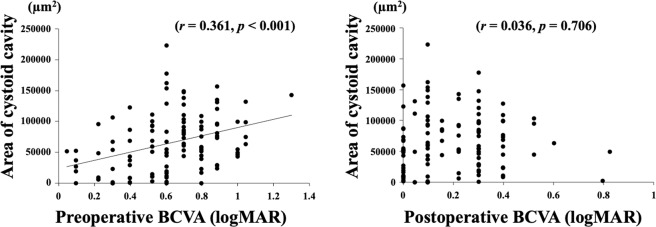
Correlation between the area of intraretinal cystoid cavity and pre- and the postoperative BCVA. The area of intraretinal cystoid cavity was significantly correlated with preoperative BCVA (**A**), but not with the postoperative BCVA (**B**).

The area of the cystoid cavity was significantly correlated with the preoperative BCVA (*r* = 0.361, *P* < 0.001) but not with the postoperative BCVA (*P* > 0.05; Fig. 3). The correlations between the summed areas of the cystoid cavities and the postoperative integrity score of the ELM, EZ, and CIZ were not significant (Table 3). The postoperative BCVA was significantly correlated with the preoperative BCVA (*r* = 0.421, *P* < 0.001), the basal MH size (*r* = 0.443, *P* < 0.001), the minimum MH size (*r* = 0.494, *P* < 0.001), the length of the ELM defect (*r* = 0.387, *P* < 0.001), the length of the EZ defect (*r* = 0.331, *P* < 0.001), and the length of the CIZ defect (*r* = 0.211, *P = *0.035) (Table 4).

**Table 3:** Results of Pearson’s correlation coefficient between the area of intraretinal cystoid cavities and the postoperative status of outer retinal layers.

Variables	Mean ± SD	*r*	*p*-value
ELM integrity score	3.96 ± 0.29	−0.119	0.419
EZ integrity score	3.69 ± 0.58	−0.178	0.225
CIZ integrity score	3.08 ± 1.13	−0.094	0.523

**Table 4:** Results of Pearson’s correlation coefficient between the postoperative visual acuity and the other variables.

Variables	*r*	*p*-value
Age (year)	0.109	0.278
Axial length (mm)	0.033	0.742
Preoperative BCVA (log MAR)	0.421	<0.001
Duration of symptom (months)	0.211	0.035
Area of intraretinal cystoid cavities (μm^2^)	0.033	0.739
Basal MH size (μm)	0.443	<0.001
Minimum MH size (μm)	0.494	<0.001
Basal - minimum MH size (μm)	0.209	0.036
Maximum retinal thickness (μm)	0.111	0.268
ELM height (μm)	0.143	0.155
Angle of fluid cuff (°)	0.021	0.831
ELM defect length (μm)	0.387	<0.001
EZ defect length (μm)	0.331	<0.001
CIZ defect length (μm)	0.211	0.035

**Figure 4:**
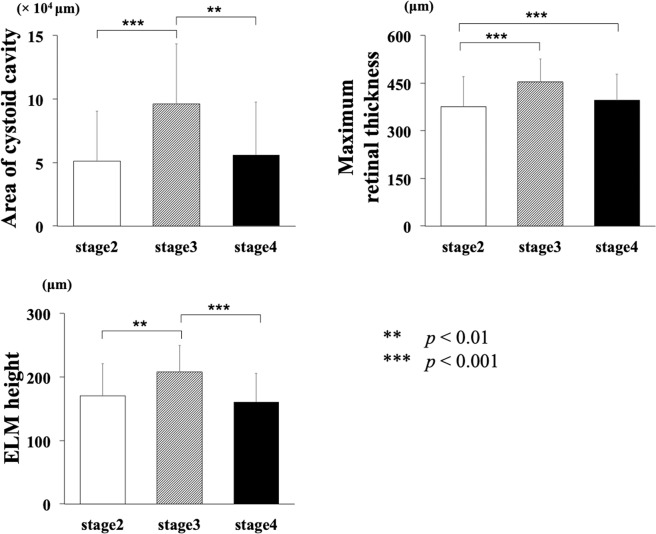
Comparison of the MH morphological features among the different MH stages. The Stage 3 MH group had the largest intraretinal cystoid area, the maximum retinal thickness, and the largest ELM height than the Stage 2 and Stage 4 MH eyes.

There were significant differences between the area of the cystoid cavities, the maximum retinal thickness, and the ELM height, and the MH stages (all, *P* < 0.001; Table 5). The area of the cystoid cavity and the ELM height were significantly larger in the Stage 3 MH group (Fig. 4).

**Table 5:** Difference of characteristics among the MH stage.

MH stage	2 (n = 42)	3 (n = 30)	4 (n = 39)	*p*-value
Age (year)	66.3 ± 6.1	66.6 ± 5.2	66.2 ± 7.0	0.972
Axial length (mm)	23.7 ± 1.2	23.6 ± 1.2	24.3 ± 1.4	0.044
Duration of symptom (month)	2.6 ± 1.8	3.1 ± 2.5	4.5 ± 3.1	0.006
Preoperative BCVA (logMAR)	0.653 ± 0.231	0.725 ± 0.181	0.505 ± 0.277	0.001
Postoperative BCVA (logMAR)	0.196 ± 0.144	0.187 ± 0.161	0.210 ± 0.204	0.863
Basal MH size (μm)	605 ± 257	738 ± 215	645 ± 251	0.079
Minimum MH size (μm)	259 ± 157	314 ± 171	279 ± 165	0.384
Maximum retinal thickness (μm)	376 ± 95	454 ± 73	396 ± 82	<0.001
ELM height (μm)	170 ± 51	208 ± 42	161 ± 45	<0.001
Angle of fluid cuff (°)	37.4 ± 4.9	36.7 ± 4.1	34.4 ± 7.3	0.054
**Area of intraretinal cystoid cavities**				
None (eyes)	3	0	2	
in INL (μm^2^)	6460 ± 8477	13373 ± 11771	8628 ± 8085	0.011
in HFL-ONL complex (μm^2^)	44681 ± 36054	82762 ± 44495	47293 ± 38883	<0.001
in all (μm^2^)	51141 ± 39459	96135 ± 47482	55920 ± 41638	<0.001

## Discussion

Our results showed that intraretinal cystoid cavities were present in 95.5% of the eyes with an idiopathic MH, and none was present in all of the eyes after the surgery. The area of intraretinal cystoid cavity was significantly correlated with the preoperative BCVA, the basal MH size, the ELM height, and the maximum retinal thickness, but not with the postoperative BCVA. The MH in the Stage 3 group had the largest intraretinal cystoid cavity area, the ELM height, and the maximum retinal thickness among the 3 stages of MHs. The postoperative BCVA was significantly correlated with the morphology determined by OCT, e.g., the MH size and the length of the defects of the microstructures of the photoreceptors.

The presence of cystoid cavities in 95.5% of the eyes preoperatively agrees with an earlier study that reported that intraretinal cystoid cavities were observed in 92.5% (37 of 40) of the eyes with a MH^7^. Our results showed that the cystoid cavities were located in both the HFL-ONL complex and the INL, and the area of the cystoid cavity in the HFL-ONL complex was significantly larger than in the INL. These results are also consistent with the results of two earlier studies^13,14^.

There have been many reports describing the relationship between the BCVA and the preoperative morphology determined by OCT in eyes with MH, e.g. the basal and minimum MH size^8,9,15^, the ELM height^16^, and the maximum retinal thickness^10^. Our results showed that the area of the cystoid cavities was correlated with the preoperative BCVA. In addition, the area was positively and significantly correlated with the difference between the basal and minimum MH size, the ELM height, and the maximum retinal thickness. In addition, eyes with larger cystoid cavity areas had greater differences between the basal and minimum MH size, higher ELM height, and longer maximum retinal thickness. These findings indicate that the retina at the edge of the MH is more elevated in eyes with larger intraretinal cystoid cavity area, and this may be related to the poor preoperative BCVA. ﻿On the other hand, the area of the cystoid cavities was not significantly correlated with the postoperative BCVA.

Sugiura *et al*. reported that the area of the intraretinal cystoid cavities were significantly correlated with the degree of postoperative metamorphopsia but not with the postoperative BCVA^13^. In addition, the areas of cystoid cavities were not significantly correlated with the integrity of the postoperative outer retinal layers. The microstructures of the outer retinal layers, viz., the ELM, EZ, and CIZ, represent the integrity of the photoreceptors, and the postoperative integrity of these layers have been identified as predictive markers of the visual outcomes in retinochoroidal diseases^17–19^ and MHs^11,12,20^. These findings suggest that the presence of the intraretinal cystoid cavities do not affect the postoperative BCVA and the restoration of any of the outer retinal layers.

In contrast to the area of the cystoid cavity, the basal and minimum MH size, and the lengths of the ELM, EZ, and CIZ defects were significantly correlated with the postoperative BCVA in our results. These results are consistent with the findings of earlier reports^16,21^. Several studies have shown that the length of the outer retinal layer defect can be a predicting factor for the postoperative vision because they are related to the MH size. On the other hand, the difference from the basal MH size to minimum MH size and the maximum retinal thickness were strongly correlated with the area of intraretinal cystoid cavities but were not significantly correlated with the postoperative BCVA. Taken together, we suggest that it is more likely that improvement of the BCVA was due to the restoration of the outer retinal layers. The degree of restoration would be more affected by the MH size and the length of the defect than the area of the intraretinal cystoid cavities. This would then account for the lack of significant correlations between the area of the intraretinal cystoid cavities and the postoperative BCVA.

A case series of serial OCTs and morphing videos demonstrated that the VMT was the initiating event for the formation of the intraretinal cystoid cavities^22^. At the early stages of MH formation, the anteroposterior tractional forces on the fovea is caused by the VMT, and foveal cystoid spaces are frequently observed^23^. Simpson *et al*. reported that the retinal stretching induced by the VMT can indeed result in a reduction in interstitial fluid pressure, with subsequent influx of water^24^.

It is believed that the cystoid spaces will expand and separate the retinal layer along with the forming of MH, and finally the intraretinal cystoid cavities are observed around the edges of the MH^25,26^. Several studies have reported on the relationships between the VMT and basal MH size and outer retinal elevation^27,28^. The basal MH size and the outer retinal elevation is increased along with the progression of primary MH due to the VMT.

There is another explanation for the formation of the intraretinal cystoid cavities. The leaked fluids may be poorly absorbed in the elevated outer retina because the retina at the fluid cuff is detached from the RPE which is responsible for the fluid transport toward the choriocapillaris.

Previous study combining the immunohistochemistry with en face OCT images demonstrated the features of the cystoid cavities^29^. The cystoid cavities in the HFL-ONL complex are large, oval, and horizontal, while those in the IPL are more vertical and cylindrical in the OCT images which is well correlated with the ﻿the radial distribution of Müller cells in the HFL-ONL complex and ﻿vertical track of Müller cells in the INL as determined by immunostaining of the normal macula. This would be related to the Z-shaped configuration of the Müller cells, and a wide zone of dehiscence with involvement of the perifoveal Z-shaped fibers may lead to large intraretinal cystoid cavities and greater elevations of the outer retina. Müller cell dysfunction would lead to the disruption of these junctions and lead to larger spacing between cells which would be observed as the cavities observed in the INL and the HFL-ONL complex. In addition, the presence of excessive fluid in the inter- and intracellular spaces could result from a dysfunction of the hydro-ionic regulation of the Müller cells.

Gass classified MHs by the extent of the vitreomacular adhesion: Stage 2, partial detachment of the posterior hyaloid; Stage 3, total detachment of the posterior hyaloid but still attached to the optic disc; and Stage 4, complete detachment of the posterior vitreous^1^. In our results, the Stage 3 eyes had significantly larger cystoid areas and higher ELM height than Stage 2 or Stage 4 MH eyes. There were 3 eyes in the Stage 2 group that had no intraretinal cyst in contrast to none in the Stage 3 group. This suggests that the intraretinal cystoid cavities around the MH are not formed at the early period after the formation of MH even if the edge of the MH has a VMT. Our results support the idea that the intraretinal cystoid cavities resulted from VMT as a consequence of the MH formation. The Stage 3 MH group had the largest area of the cystoid cavity which implies that the metabolic functions of Müller cells may remain altered after the release of the VMT and the formation of a full-thickness MH. On the other hand, the area of the cystoid cavity and the ELM height in Stage 4 MH group were smaller than those in Stage 3 eyes. Yun and associates examined the morphologic characteristics of eyes with a chronic MH^30^. They reported that all of the eyes with a chronic MH were at Stage 4, and intraretinal cystoid cavities were observed less frequently in eyes with a chronic MH compared to eyes at the acute Stage 4^30^. Our results showed that 2 eyes in the Stage 4 group had no intraretinal cystoid cavity and the area of the intraretinal cystoid cavities were smaller. These finding corroborate their report.

There are limitations in this study. First, this was a retrospective study, and we did not evaluate the longitudinal changes of the OCT parameters including the intraretinal cystoid cavities. It was not determined when the intraretinal cystoid cavities developed and how they progressed over time. Second, the area of intraretinal cystoid cavity was measured manually in the OCT B-scan images. We measured the mean values of the area on the perpendicular images with OCT radial scans and confirmed that the horizontal and vertical images had the same cystoid cavities. However, some studies have evaluated the cystic areas in the en face OCT images, and similar measurements in our patients would probably lead to a more precise assessment of the cystic areas^29,31^. Third, the follow-up period was relatively short. Fourth, metamorphopsia and its relationship between the morphology of the MH were not evaluated. Further prospective longitudinal studies with automated calculations of the morphological changes and an evaluation of metamorphopsia will be necessary to validate the relationship between the intraretinal cystoid cavities and the pre- and postoperative BCVAs.

In conclusion, we found that intraretinal cystoid cavities were present in 95.5% of the eyes with an idiopathic MH before vitrectomy, and all were resolved after the vitrectomy. The presence of intraretinal cystoid cavity is correlated with the preoperative vision but not the postoperative vision and the restoration of any outer retinal microstructures.

## Methods

### Ethics statement

This was a retrospective, cross sectional, single center study, and the procedures used were approved by the Ethics Committee of the Nagoya University Hospital, Nagoya, Japan. The study was performed at the Nagoya University Hospital, and the procedures used conformed to the tenets of the Declaration of Helsinki. Written informed consent was obtained from all patients.

### Subjects

We reviewed the medical records of patients with a unilateral, idiopathic MH who had undergone successful MH closure by vitrectomy at the Nagoya University Hospital from January 2013 to June 2018. The inclusion criteria were the availability of high quality spectral domain-OCT (SD-OCT) images (Spectralis; Heidelberg Engineering, Heidelberg, Germany) and the presence of a Gass Stage 2, 3, or 4 idiopathic full-thickness MH as determined by OCT. All patients underwent comprehensive ophthalmic examinations including measurements of the BCVA, intraocular pressure, and axial length. In addition, the retinas were examined by slit-lamp biomicroscopy, fundus photography, and OCT before and 12 weeks after the vitrectomy. The Snellen visual acuities were converted to the logMAR units to create a linear scale of the BCVAs.

The exclusion criteria were previous or coexisting ocular conditions, e.g., mature cataracts, high myopia (axial length >27.0 mm); traumatic MH, recurrent MH, other retinal disease, and prior vitrectomy.

### Analyses of OCT images

The preoperative images of the retina obtained by SD-OCT B scans through the center of the MH were analyzed. All images were adjusted to correct for the difference of the vertical and horizontal ratios to be the same proportion. The basal MH size, the minimum MH size, the maximum retinal thickness (Fig. 1A), the ELM height, and the angle of the fluid cuff were measured in the vertical and horizontal OCT images (Fig. 1B). The average of two measurements was used for the statistical analyses. The distance between each edge of the outer retina was defined as the defect length, and the lengths of the ELM, EZ, and CIZ defect were measured in the image in which the basal MH size was the largest (Fig. 1C).

The area of the intraretinal cystoid cavity was measured with the ImageJ software (National Institutes of Health, Bethesda, Maryland, USA; available at https://imagej.nih.gov/ij/). The analysts were two of the authors (K.G., T.I.) who were masked to the other clinical information. The analyst traced the outline of the intraretinal cystoid cavities using the ImageJ program by freehand drawing and summed all of the values in one cross-sectional image passing through the fovea (Fig. 1D). If no intraretinal cystoid cavities were observed, the area of the intraretinal cystoid cavity was designated as zero.

The OCT images obtained at 12 weeks postoperatively were used to determine the integrity of ELM, EZ, and CIZ for a 1,000-mm diameter area surrounding the fovea. The microstructures were graded on a 4-point scale: 1, line not visible; 2, line disrupted by 0.200 mm; 3, line disrupted by >200 mm; and 4, continuous line^17^.

### Surgical techniques

A standard three-port pars plana vitrectomy was performed using the Alcon Constellation 23- or 25-gauge (G) system (Alcon Laboratories, Inc., Fort Worth, TX, USA). Core vitrectomy was performed, and if necessary, a posterior vitreous detachment was created by suction with a vitrectomy probe around the optic nerve head. Then, the ILM was peeled from the retina circumferentially with ILM-peeling forceps in all cases. A peripheral vitrectomy with shaving of the vitreous base was performed, and fluid-air exchange was done followed by the injection of 20% sulfur hexafluoride or 12% perfluoropropane into the vitreous to tamponade the retina. The patients were instructed to maintain a prone position until a closure of the MH was confirmed by OCT.

Cataract surgery was performed on all 95 phakic eyes and a foldable acrylic IOL was implanted into the capsular bag.

### Statistical analyses

The values are presented as the means ± standard deviations. Pearson correlation coefficients were calculated to determine the significance of the associations between the areas of the intraretinal cystoid cavity and other variables. One-way analysis of variance (ANOVA) was used to evaluate the differences in the variables among the MH stages. All statistical analyses were performed using the Statistical Package for Social Sciences for Windows 21.0 (SPSS Inc, Chicago, Illinois, USA).
